# Developing occupational therapists’ capabilities for decision-making capacity assessments: how does a support role facilitate workplace learning?

**DOI:** 10.1007/s40037-020-00569-1

**Published:** 2020-03-31

**Authors:** Janine Matus, Sharon Mickan, Christy Noble

**Affiliations:** 1 Allied Health, Gold Coast Health, Gold Coast, Australia; 2 Faculty of Health Sciences & Medicine, Bond University, Gold Coast, Australia; 3 School of Medicine, Griffith University, Southport, Australia; 4 Faculty of Medicine, The University of Queensland, Herston, Australia

**Keywords:** Educational techniques, Learning, Occupational therapy

## Abstract

**Introduction:**

Healthcare practitioners are required to develop capabilities in an effective and efficient manner. Yet, developing capabilities in healthcare settings can be challenging due to the unpredictable nature of practice and increasing workloads. Unsurprisingly, healthcare practitioner development is often situated outside of practice, for example in formal teaching sessions. Supporting practitioners to develop capabilities through engagement with day-to-day practice, whilst advantageous in terms of authenticity and being highly valued, remains a key challenge for healthcare educators. This qualitative interview study aimed to explain, from the learner’s perspective, how a dedicated support role develops occupational therapists’ capability to contribute to decision-making capacity assessments.

**Methods:**

Individual semi-structured interviews were conducted with a purposive sample of 12 occupational therapists. Informed by workplace learning theory, interview transcripts were analyzed using thematic analysis process.

**Results:**

Participants provided rich descriptions of how they developed in their capability to engage in decision-making capacity assessments. Participants reported that their learning was facilitated by the dedicated support role in three key ways: 1) structuring a journey of learning, 2) providing tailored guidance, and 3) fostering a supportive learning environment.

**Discussion:**

Participants valued the authentic workplace learning opportunities afforded by the dedicated support role. Findings suggest that capabilities, such as decision-making capacity assessment, can be developed through practice when enriched by a dedicated support role. However, further research examining the sustainability and transferability of this model and its application to other capabilities are warranted.

## Introduction

It is both important and challenging for healthcare practitioners to develop capabilities in an effective and efficient manner while providing excellent care to their clients. Moreover, development of capabilities is complex. For example, being capable, defined as “integration of knowledge, skills, personal qualities and understanding used appropriately and effectively” (p.2) [1], means that practitioners are able to integrate their capabilities in both familiar and unfamiliar settings, and/or under focussed or complex and changing conditions [2]. Increasingly, the importance and value of learning by engaging in work-related tasks has been emphasized [3–5] and theoretical models explaining how practitioners learn are being advanced [4, 6]. In one model, medical residents transform information into knowledge through an active process of interpretation, construction of meaning and reflection [6]. Another model of experience-based learning among medical students emphasizes learners’ active and supported participation in work tasks as core conditions for learning in the workplace [4].

Although learning through practice offers an effective means to support the development of capabilities, many clinicians tend to rely on pedagogical practices situated outside of practice, such as workshops or formal teaching sessions, which are the most common formats for continuing professional development [7]. Adopting this approach is understandable as workplace learning opportunities can be high stakes, unpredictable, or hampered by excessive workloads [3]. Moreover, it can be difficult to plan and facilitate authentic learning experiences, which provide rich opportunities to develop capabilities, within complex, varied and unpredictable practice settings. To address this challenge, Billett’s workplace learning theory [8, 9] can help explain how learning occurs through clinical practice and assist in identifying opportunities for how practice-based learning might be augmented.

### An overview of decision-making capacity assessment

For the purposes of this study, performing decision-making capacity assessments (DMCAs) was identified as a capability. Essentially, a DMCA assesses an individual’s ability to understand and appreciate the nature and effect of a decision, freely and voluntarily make the decision, and communicate the decision [10–12]. Healthcare practitioners are increasingly encountering situations where assessing a client’s decision-making capacity plays an integral role in the management of their care [13–15]. Concerns about capacity may be triggered when clients make choices that put their health, assets, property, self or others at significant risk of harm, and they appear to lack an appreciation of the reasonably foreseeable risks and consequences associated with those choices [12, 16].

DMCAs are considered to be capabilities for the following reasons. Firstly, practitioners need to know that DMCAs are multifaceted, time consuming, intrusive, and may have serious and far-reaching legal, ethical and practical implications for clients [17]. Therefore, best practice is that they are undertaken only if: 1) there is a need for a decision to be made, 2) there is a significant risk of harm, 3) all reversible conditions which may impact on decision-making have been ruled out, and 4) all less restrictive options for managing the risk of harm have been exhausted [18]. Secondly, DMCAs are interprofessional and should involve input from medical and allied health professionals who can undertake holistic evaluations of the psychological, social, cognitive, functional and medical factors, as well as a direct assessment of the person’s decision-making process [18, 19]. Finally, occupational therapists’ contributions to DMCAs are valued [11, 18–20], particularly where there is a question about an adult’s ability to make decisions about personal matters such as discharge destination or care arrangements [16]. In these situations, occupational therapists can provide supporting evidence regarding a client’s functional abilities, limitations and associated risks of harm, and implement interventions to maximize independence and minimize the risks of harm. Delivering these assessments and interventions requires the integration of specialized and non-routine and non-procedural knowledge and skills, and the development of an extended professional identity which includes contributing to DMCAs. Additionally, occupational therapists need to apply their capability in unfamiliar and varied contexts where each case is unique.

### Developing healthcare professionals’ capabilities in decision-making capacity assessment

Several studies have demonstrated that medical and allied health professionals often lack the readiness required to perform DMCAs [13, 19, 21–25]. These findings highlight the need for additional education, training and support. However, existing research tends to focus on implementation strategies, that is, how to best implement DMCA in healthcare settings and the main pedagogic practice adopted, as part of the implementation, was workshops [18, 19]. Whilst their evaluation of the workshops reported an increased understanding of concepts relating to DMCAs, workshops may not fully meet learning needs as they are often decontextualized from authentic practice experiences [26]. Indeed, a further evaluation found that the combination of dedicated resources, timely access to education and mentoring enhanced health professionals’ self-reported ability to confidently, competently and collaboratively conduct DMCAs [25]. However, practical strategies for educators are absent.

In 2013, the occupational therapy department of a large metropolitan state-funded hospital allocated temporary funding for a senior level occupational therapist to develop and implement a dedicated Decision-Making Capacity Assessment (DMCA) Support Role for occupational therapists. The purpose of the DMCA Support Role was to facilitate occupational therapists’ development of knowledge, skills, attitudes and confidence required to contribute to DMCAs. A range of pedagogical practices were implemented as part of a structured workplace practice curriculum. These included direct instruction (1:1 and small group sessions, work shadowing and joint assessments), guided learning (personalized advice and ‘hands-on’ guidance to work through each step of capacity cases), and resources to support learning and clinical reasoning (flowcharts, interview guides and documentation examples). However, it is not clear how the DMCA Support Role may have contributed to occupational therapists’ learning.

Whilst other studies have begun to explore how specific skills, such as prescribing, are developed in the workplace [27, 28], a comprehensive understanding of how the development of capabilities can be facilitated is lacking. This understanding will assist with generating practical strategies for practice-based educators to support the development of other capabilities through practice. Using decision-making capacity assessments as an example, the research described here explores how capabilities, from the learners’ perspective, i.e. occupational therapists, were developed through the practice-based pedagogies enabled by a dedicated support role.

### Aim

This qualitative interview study, informed by workplace learning theory, aimed to explain from the learner’s perspective how a dedicated support role develops occupational therapists’ capability to contribute to decision-making capacity assessments. A secondary goal was to share our understanding of how capability development can be facilitated in the workplace.

## Methods

This qualitative interview-based study, following a constructivist approach [29], sought to provide clarification [30] on how a dedicated support role contributes to capability development in the workplace. To explain this complex process, we drew on Billett’s workplace learning theory [31].

### Theoretical framework

Workplace learning theory explains how practitioners learn in the workplace and can be understood as an interdependent process, where meaningful learning is dependent on the interaction between the available work activities and experiences (i.e. affordances), and the way learners choose to engage with them (i.e. engagement) [32]. The quality of learner engagement with workplace affordances is dependent on their readiness, that is: what they know, can do, and value [32]. Learners can be supported to develop different types of knowledge, including conceptual knowledge (what individuals know), procedural knowledge (what individuals can do) and dispositional knowledge (what individuals value) through establishing a structured practice curriculum and implementing a range of practice pedagogies. Expert guidance can enable individuals to extend their scope of learning beyond what they could achieve through their own efforts [32]. These considerations are important to understand how dedicated support roles contribute to workplace capability development through augmenting the interplay between contextual considerations (i.e. affordances) and individual engagement.

### Context

This study was conducted in a large Australian tertiary hospital (750 inpatient beds). Ethical approval for this study was obtained from the Human Research Ethics Committee of the Gold Coast Hospital and Health Service. HREC/16/QGC/324. This research was carried out in accordance with the Declaration of Helsinki. Approximately 65 occupational therapists work within an occupational therapy department that services acute care and rehabilitation settings. Across these settings, occupational therapists receive approximately 350 referrals per year to assist with gathering information to inform DMCAs. These referrals represent a small proportion of total referrals but often consume a disproportionate amount of time due to their complexity. Prior to the establishment of a part-time (0.4 FTE) DMCA Support Role in 2013, there was no specific education or support available to guide occupational therapists’ practice in this area. The DMCA Support Role was established and championed by managers, who allocated a proportion of existing funding to it, with the goal of improving occupational therapists’ capabilities and contributions to DMCAs. It was separated from line management structures and not involved in performance evaluation or management of staff. The persons employed in the DMCA between 2013 and 2017 also worked in other clinical and education roles in the same organization.

### Participants

Occupational therapists were eligible for inclusion in the study if they had accessed the DMCA Support Role for a period of at least 6 months. Purposive sampling [33] was used to select participants who were likely to provide rich information and a diverse range of perspectives about how the DMCA Support Role influenced their learning [34]. We purposively sampled a mixture of recently graduated, junior and senior occupational therapists, and those working in different practice settings including acute care and rehabilitation. Eligible participants were invited to participate via an emailed letter including a participant information sheet and consent form. Invitations and responses were managed by an independent administrative officer to minimize the influence of social desirability bias on the decision of potential participants to take part. All participants provided informed consent. Participants were recruited until sufficiency was achieved, that is, answering our research question and providing rich insights ensuring transferability to other contexts [35].

### Data collection instrument

Billett’s workplace learning theory [8, 32] informed the development of the interview guide. The interview guide included questions about participants’ personal history of learning about and developing knowledge and skills to contribute to DMCAs, their experiences of engaging with the DMCA Support Role and their perceptions of the influence this support had on their practice. Questions were semi-structured to elicit a broad range of responses.

### Procedure

Individual face-to-face interviews were conducted in early 2017 by two independent senior neuropsychologists working in the same hospital. These interviewers were not involved in the development or implementation of the DMCA Support Role but nevertheless had an in-depth understanding of the complexity of DMCA and an awareness of the purpose of the DMCA Support Role, and thus were able to ask appropriate probing questions. Both interviewers were thoroughly briefed regarding the aims of the research project. Interviews were MP3 recorded, transcribed verbatim by a professional transcribing service and de-identified. Following the completion of their face-to-face interview, participants were also invited to confidentially add any additional comments via a secure online platform.

### Data analysis

Thematic analysis was undertaken using a recursive process and following the phases described by Braun and Clarke [36]: 1) becoming familiar with the data by reading the transcripts, 2) generating initial codes, 3) grouping the codes, based on workplace learning theory, and developing themes, 4) identifying patterns in the data through participants ‘indigenous categories’ [37], and 5) defining and naming the themes. Specifically, one author (JM) read and openly coded all transcripts, identifying keywords, concepts and recurring patterns of meaning in the data. Another author (CN) also openly coded a sub-sample of four transcripts. A small number of discrepancies were resolved through discussion and the resulting codes were then applied to all transcripts by JM for consistency. The codes were collated into preliminary semantic themes which were then reviewed and further organized and synthesized using workplace learning theory [32] as a frame of reference. Throughout this process, mind maps were used to conceptualize relationships between themes [36]. All the authors were involved in this final phase of analysis to maintain reflexivity, challenge assumptions and to check the validity to help maintain trustworthiness, credibility and accountability of the findings [38]. The authors participated in regular meetings which helped them to develop their understanding of workplace learning theory and how it applies in the context of this study. Ultimately, all the authors agreed on the final themes. These themes were then also checked and confirmed by a sub-sample of interviewees to be accurate representation of their perspectives.

## Results

Of the 21 occupational therapists who were invited, 12 agreed to participate while two were on leave and nine did not respond to the invitation. The sample consisted of one male and 11 females. The median years of experience working as an occupational therapist in hospital-based settings was 3.5 years (range 1–15 years), which is representative of the demographics of the occupational therapy department (Tab. 1). The median length of interviews was 24 minutes (range 18–31 minutes).

**Table 1:** Demographics of study participants (*n* = 12)

*Gender*	
Female	11 (92%)
Male	1 (8%)
*Years of experience working as an OT*	
<2	1 (8%)
2–5	5 (42%)
5–10	5 (42%)
>10	1 (8%)
*Clinical area*^a^	
Acute care	10 (84%)
Rehabilitation	7 (58%)

*OT* occupational therapist

^a^Most participants had worked in both acute care and rehabilitation.

Participants provided rich descriptions of their experiences of being involved in DMCAs and how their capabilities had changed over time. All participants found DMCAs to be complex and challenging, and felt inadequately prepared as they had not previously learned the relevant knowledge and skills, for example at university. The participants then described their experiences of engaging with the DMCA Support Role. Overall, all participants reported very positive impressions of the DMCA Support Role and the influence it had on their learning. Based on our analysis, three inter-related themes relating to how the Support Role facilitated their learning in a workplace setting were identified. They included: 1) structuring a journey of learning, 2) providing tailored guidance, 3) fostering a supportive learning environment. Tab. 2 outlines these three themes, suggested strategies for educators and supporting illustrative quotes. Each theme will now be described in sequence, with italicized quotes providing additional contextual evidence from participants.

**Table 2:** Strategies identified for supporting learning in the workplace

Themes	Illustrative quotes	Strategies
Structure a journey of learning	*“I first started having these initial education sessions to set the scene, which really supported me and gave me the base knowledge to understand my role, then getting resources to follow up and read through … then consolidating that through practice, with constant support. It’s the ongoing support that’s the most important.” (OT8)* *“I think initially, having someone specifically support you though that process for at least the first couple of times is really valuable.” (OT12)* *“Now it’s only when I have the really, really complex patients that I seek out a bit more help.” (OT8)* *“Now I realize that when they say they’re looking into capacity, it’s not this stressful thing. It’s work as per normal but just some additional information gathering.” (OT6)*	Ask about learners’ prior experience and knowledge; match the type and amount of guidance to their needs i.e. assess learner readiness. Maximize opportunities for learners to be actively involved in authentic work tasks. Provide ongoing progressive support which is most intensive for the earliest and most complex tasks. Scaffold explicit links between new and existing knowledge; current and previous experiences. Start by giving learners responsibility for less complex steps while assisting with more complex steps of tasks.
Provide tailored guidance	*“She’ll ask you, ‘what have you been doing so far?’ or ‘what things have you tried so far?’’’ (OT3)* *“(The person in the Role) would come to me and say ‘this is what’s expected of you’, or ‘here’s what you do’ … she will give you very clear direction and instructions.” (OT9)* *“The education sessions … helped guide me through the flowcharts and the resources that we already have, and really making it about the clinical reasoning and the justification for why we’re doing what we’re doing.” (OT8)* *“It’s definitely been extremely helpful just to get another perspective and support in terms of making decisions about, well, what assessments might be helpful and how they can be interpreted … and maybe help you consider some things that you haven’t thought of yet.” (OT10)* *“A couple of times (the Role) actually came and did the interview with me and we co-did the interview. I’m quite a visual learner and actually seeing someone do it was so powerful.” (OT6)* *“I think a lot of learning has come through the ability to talk through my clinical reasoning process, be challenged, and reflect on those as well.” (OT4)*	First ask learners what they think they should do before offering advice or suggestions. If required, provide some direct instructions and specific advice about what to do and how to do it, but also provide in-depth explanations of why to do it. Explicitly articulate your clinical reasoning and decision-making process. Offer opportunities for learners to debrief about challenging work tasks and gain a second perspective. Develop resources such as guidelines, flowcharts, templates, worked examples, videos and simulations. Role model and demonstrate key knowledge, skills, attitudes and values e.g. during joint clinical sessions. Provide constructive feedback about learners’ knowledge, practical skills and clinical reasoning. Facilitate structured reflection on practice and ask learners to summarize “key take-home messages” of what they have learnt from each experience.
Foster a supportive learning environment	*“Through those initial education sessions, I built rapport with the (person in the) Role, which I felt was really supportive and I felt more than comfortable calling her to be able to help me out with any particular hard cases.” (OT8)* *“It’s not expected that everyone should have this knowledge; it’s really acknowledged as a specialty area …” (OT4)* *“I was finding that if there was a question that I had, timeliness as far as the response goes has always been fantastic.” (OT12* *“(The Role) is really removed, so people aren’t worried that feedback is related to performance or contracts or anything. It’s a really safe way to learn, make mistakes, get feedback, get supported, do joint sessions.” (OT4)*	Build rapport with learners, e.g. by finding out about their interests and previous experiences. Normalize the challenges inherent in complex areas of practice and clarify expectations about proficiency. Be approachable and actively promote availability, e.g. by regularly visiting teams in their work area. Wherever possible, provide flexible and timely support when it is needed. Where possible, separate learning support from performance evaluation processes.

### Structuring a journey of learning

All participants noted that by engaging with the DMCA Support Role, their learning experiences were structured and sequenced in a progressive way. Most participants reported having no or limited knowledge about DMCAs prior to engaging with the DMCA Support Role, and those who had worked in the department prior to the Support Role being established recalled a lack of information, support and resources. Following an introductory education session, participants described being given opportunities to work through a series of authentic DMCA cases with ongoing graded support. Some participants recalled receiving more intensive support for their first few DMCA cases, followed by decreasing support as their capability and confidence increased over time. However, even more experienced participants continued to access support for complex cases.

Half of the participants reported that the DMCA Support Role supported them to make explicit links and associations between new and existing knowledge. For example, they were asked to reflect on their previous experiences of contributing to DMCAs and to consider the similarities and differences between cases. Further, they were guided to compare the scope of their role in DMCA with the more general occupational therapist role in acute care and rehabilitation settings, and to consider how their knowledge and skills were transferable between these contexts.

### Providing tailored guidance

Participants described how the DMCA Support Role provided them with different types of guidance depending on their needs and preferences. Most participants described receiving information and direct instructions about what to do and how to do it, alongside in-depth explanations of the reasoning behind why they should be doing it, i.e. in relation to best practice principles and professional values.

In addition to receiving information and instructions directly from the DMCA Support Role, most participants reported that they benefited from having access to resources and artefacts such as written guidelines, flowcharts, case studies, templates and worked examples. While some participants valued direct instructions, others preferred to utilize the Support Role as more of a sounding board to talk through their observations and clinical reasoning, gain a second perspective or seek confirmation of being on the right track. This is illustrated by the following quote:

*The advice that I received from the Role tended to be more confirming, … it kind of extended on what I’ve already been thinking … to take you through the next step and to that next level of considering what’s happening. [OT11]*

Most participants reported learning from demonstration and modelling of how to conduct an interview, write a report or communicate recommendations with other members of the team. Participants appreciated opportunities to be involved in joint sessions, during which they could observe more complex or unfamiliar steps while still maintaining an active role in the assessment process. Some participants valued receiving feedback and support to reflect on their practice, which helped them to continually improve their knowledge and skills.

Of note, there was variability in how participants engaged with the DMCA Support Role. While some actively sought out support, others were more passive in waiting for the Support Role to initiate contact. A key factor which seemed to influence this was having an appreciation of the seriousness of DMCAs and valuing what occupational therapists can contribute to the management of these cases.

### Fostering a supportive learning environment

In addition to receiving tailored guidance, participants emphasized the importance of having a supportive and trusting relationship with the DMCA Support Role, which allowed them to feel comfortable asking for help, participating in joint assessments and receiving constructive feedback on their performance along with plans for subsequent improvement. This was facilitated by building rapport, being approachable, normalizing DMCA as a challenging area of practice and separating the DMCA Support Role from line management and performance evaluation processes.

Another important factor reported by most participants was knowing that the Support Role is consistently available, responsive and able to provide timely support when it was needed. This is illustrated by the following quote:

*I feel very supported with (the Role). I know that as soon as I get one that may be even looking like (a capacity case), I know that I’m going to have as much support as I need. [OT5]*

In these ways, the participants valued being supported as they developed their capability in conducting DMCAs.

## Discussion

Three themes emerged about how participants perceived that the DMCA Support Role influenced their learning and contributed to capability development. These included: 1) structuring a journey of learning, 2) providing tailored guidance, and 3) fostering a supportive learning environment. Moreover, this paper provides practical examples of how workplace learning theory [8] can explain and identify strategies used to support the learning of capabilities in a healthcare setting. Some examples of these strategies are outlined in Tab. 2, in relation to the three themes and supported by illustrative quotes.

Firstly, a practice curriculum can be progressive even when client presentations are unpredictable [39]. Our findings suggest that the DMCA Support Role, whilst not being able to sequence learners’ access to DMCA cases in a predetermined order, made important contributions to the occupational therapists’ learning in a graded way. In particular, by providing more intensive support during the first few cases, by giving learners increasing responsibility for more complex aspects of clients’ care, and by transitioning from direct to indirect support as their capabilities and confidence increased. Moreover, our findings suggest that the DMCA Support Role supported occupational therapists’ learning by formulating the practice curriculum based on an assessment of their readiness to engage in DMCAs (i.e. what they knew, could do and valued) [32]. This finding is important because effective workplace learning is dependent on learner engagement. Overall, these findings, whilst reinforcing those made by others such as Chen et al. [40], are novel because they highlight the importance of assessing learner readiness and appropriately sequencing workplace learning opportunities, in the context of a safe learning environment, when developing capabilities. Secondly, our findings suggest that it was the variety of practice pedagogies (e.g. direct guidance, role modelling, meaningful artefacts) tailored to individual learners’ needs that assisted with developing capabilities. This finding is important because when developing new capabilities in healthcare settings, organizational responses tend to be to offer one-off education sessions situated outside of practice, such as lectures and workshops.

While existing literature has demonstrated the effectiveness of providing guidance in the context of real-life work tasks, our findings suggest tailored guidance that is provided in a timely and responsive manner during a learner’s first few DMCA cases may be more important for the development of capabilities than later on when they have more experience. Although there are financial resource implications associated with providing intensive and tailored support for individual learners, this could be offset by reducing the amount of resources invested into providing didactic lectures and workshops. These traditional educational interventions are often decontextualized from real-life work activities and may afford limited opportunities for active participation [5], resulting in limited uptake and translation of new knowledge into practice.

Finally, in line with previous research [41], this study confirms that when developing capabilities, building a trusting relationship between the DMCA Support Role and the occupational therapist was paramount. In particular, it was the combination of expertise of the DMCA Support Role, meaningful feedback and facilitating reflection on practice within the context of a supportive relationship [3, 5, 42, 43]. An important feature which facilitated this successful relationship was separating the DMCA Support Role from performance evaluation and line management responsibilities, thereby reducing learners’ anxiety about disclosing gaps in their knowledge and participating in joint sessions.

### Strengths and limitations

This study investigated how capabilities can be developed in the workplace when supported by a dedicated support role. A key strength of this study is that it offers a fresh perspective on practical strategies to support learning through practice which are theory informed. The final themes evolved through regular reflexive discussions between authors which led to a deeper understanding of how learning takes place. Author CN challenged JM to consider how workplace learning theory can illuminate and explain the processes of capability development as influenced by a dedicated support role [44]. Whilst JM in turn possessed an in-depth and rich understanding of the context and data ensured that the findings were confirmable [45] i.e. based on the participants’ perspective.

As with all studies, there are limitations to our work. First, this was a single institution study using a small sample of occupational therapists which is not necessarily representative of the wider population of occupational therapists or other healthcare practitioners. However, using workplace learning theory as an analytical framework enabled a comprehensive understanding of the key features required for capability development [46]. Secondly, the findings of this study were undoubtedly influenced by factors relating to the local environmental context and characteristics of the person who occupied the DMCA Support Role, who was also a member of the research team. Yet potential transferability is enhanced by rich descriptions and purposive sampling [47, 48]. Also, to address the potential for positive bias, participant recruitment and interviewing were conducted by independent investigators, and participants were assured confidentiality.

Third, participants provided self-reports of factors influencing their learning. These self-reports may not be an accurate reflection of their actual learning or changes in their capabilities. However, they are important accounts because they represent the learners’ lived experience of engaging with the DMCA Support Role. This was an initial study to explore participants’ perceptions of how strategies implemented by the DMCA Support Role had influenced their learning.

### Future directions

Whilst this study provides important insights into how a support role can facilitate the development of capabilities in clinical settings, there would be value in conducting similar studies to examine the development of other capabilities within different clinical settings, such as end-of-life care outside of traditional palliative care settings [49]. Follow-up observational studies would be helpful to specifically map the pedagogic practices used and examine the influence of different affordances, such as social and contextual factors [8]. Additionally, further research could evaluate changes in learners’ knowledge, skills, attitudes, and resulting changes in their clinical practices [50]. Finally, there would be value in evaluating the financial sustainability of the model.

## Conclusions

This paper provides a practical example of how workplace learning theory can identify strategies for supporting learning in healthcare settings. To develop capabilities in a healthcare setting, learners benefit from receiving ongoing tailored guidance to actively participate in a structured curriculum of authentic work-based interactions and activities. Further, learners benefit from receiving feedback and being guided to reflect on their practice within a safe and supportive learning environment. Although the findings of this study relate to a specific context, the workplace learning strategies implemented by the DMCA Support Role may have relevance to other settings where learners need to develop capabilities.
